# Transcatheter valve-in-valve-in-valve replacement in tricuspid position in a patient with pre-existing permanent dual-chamber pacemaker

**DOI:** 10.1007/s00392-021-01842-x

**Published:** 2021-04-28

**Authors:** Philipp Lake, Elmar W. Kuhn, Victor Mauri, Sascha Macherey, Julia Kaliba, Stephan Baldus, Christian Frerker, Tobias Schmidt

**Affiliations:** 1 Department of Internal Medicine III, University Hospital of Cologne, Kerpener Straße 62, 50937 Cologne, Germany; 2 Department of Cardiothoracic Surgery, Heart Center, University Hospital of Cologne, Cologne, Germany; 3 AngioConsult GmbH, Speyer, Germany; 4 Department of Internal Medicine II, University Hospital of Schleswig-Holstein, Campus Lübeck, Germany

**Keywords:** Tricuspid valve, Valve-in-valve replacement, Transcatheter valve replacement, Pacemaker dysfunction

## Abstract

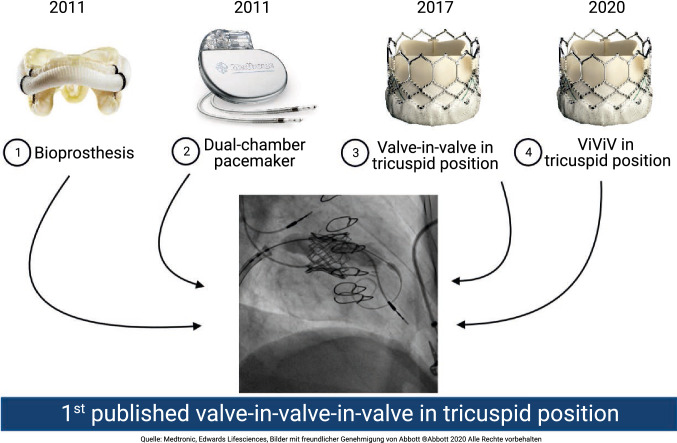

Sirs:

Patients after surgical tricuspid valve repair or replacement frequently need repeat surgery for structural valve degeneration. However, redo surgery has been associated with high perioperative mortality and morbidity (~ 26%) [1, 2]. Transcatheter valve-in-valve replacement in degenerated surgical bioprosthetic valves in tricuspid position has been previously described as an alternative to redo surgery [3–7]. Here we report for the first time a redo transcatheter valve-in-valve-in-valve procedure in a patient with a dysfunctional transcatheter valve-in-valve prosthesis (Edwards SAPIEN 3, 29 mm) in a prior surgically implanted prosthesis (St. Jude Medical, 31 mm) in tricuspid position.

A 38-year-old female patient with a complex past medical history including multiple open-heart surgeries presented with severe dyspnea and recurrent syncope. The patient had a history of intravenous drug abuse with recurrent infective tricuspid valve endocarditis, being in a substitution program at the time of presentation. Additional diagnoses included hepatitis C and chronic obstructive pulmonary disease (FEV_1_ 63% of the predicted value).

At the age of 13 the patient had undergone surgical patch closure of a congenital atrial septal defect (ASD). Because of infective tricuspid valve endocarditis, surgical reconstruction (de Vega) was performed in 2006 with removal of a vegetation and partial removal of the anterior and septal leaflet. Recurrent tricuspid valve endocarditis in 2011 required surgical valve replacement with implantation of a St. Jude Medical 31 mm bioprosthesis, followed shortly afterwards by the implantation of a dual-chamber pacemaker (Medtronic Relia REDR01) due to complete heart block with the ventricular lead positioned through the prosthesis. In 2017 a first transcatheter valve-in-valve replacement (Edwards SAPIEN 3 29 mm) was performed due to structural degeneration of the surgically implanted valve, thereby trapping the ventricular pacemaker lead between the SAPIEN and the surgically implanted SJM prosthesis.

At time of presentation, echocardiography showed relevant stenosis (peak/mean gradient 12.7/5.5 mmHg) and a mild regurgitation of the SAPIEN prosthesis. Right heart dimension and function were normal (RV basal diameter 34 mm, RA area 19.5 cm^2^), left ventricular ejection fraction was preserved. Further diagnostic workup with 4D-computer tomography ruled out leaflet thrombosis of the SAPIEN 3 and confirmed valve dysfunction as cause of the stenosis with evidence of absent and impaired motion of the right cranial and left cranial leaflet, respectively, which was in line with the echocardiographic findings (Fig. 1). The laboratory showed no relevant changes. Pacemaker interrogation revealed persistent complete AV block with absolute pacemaker dependency.

**Fig. 1:**
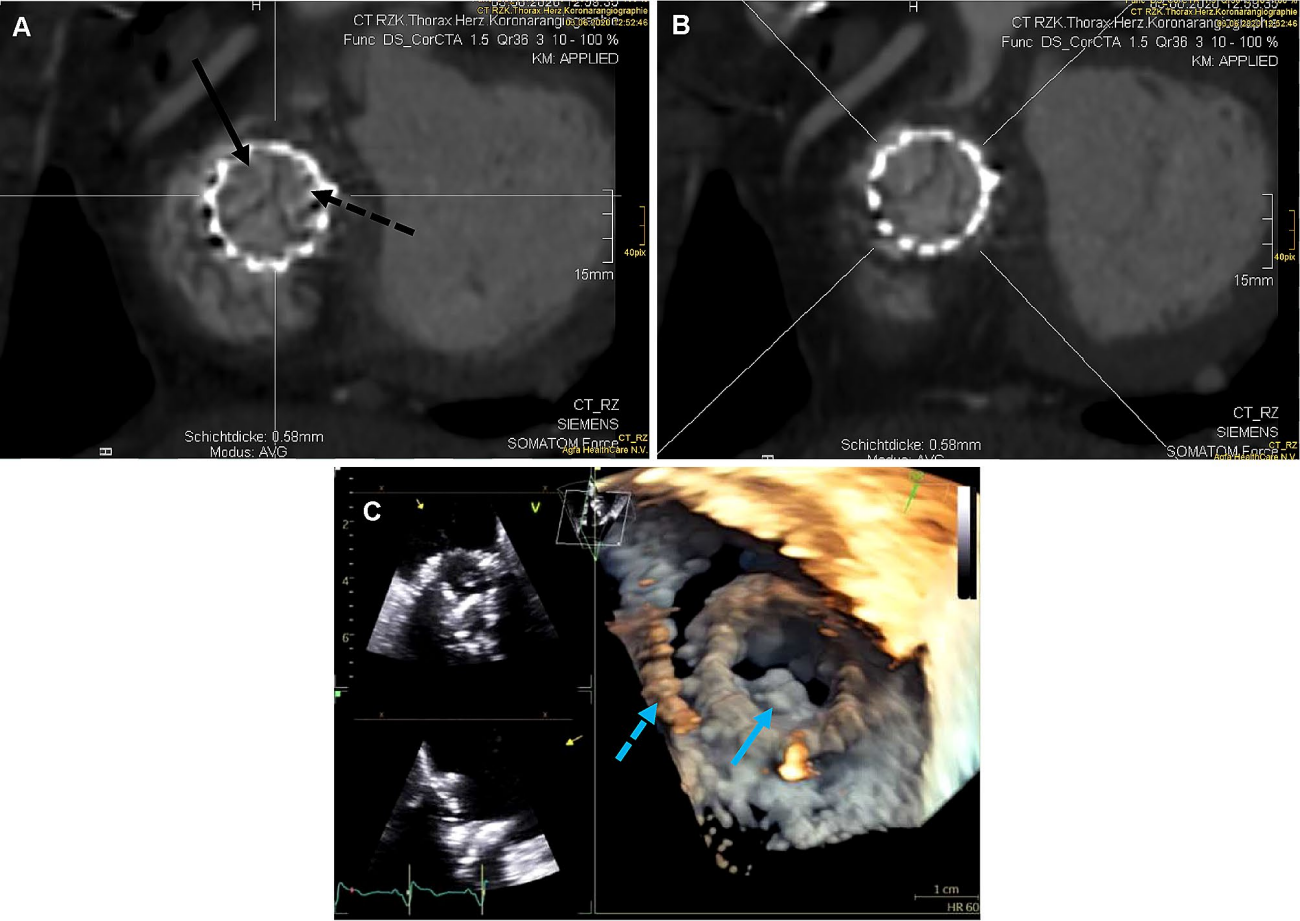
4D-CT and transesophageal echocardiography showing the dysfunctional transcatheter implanted valve-in-valve prosthesis in tricuspid position. **a** During ventricular systole the leaflets of the bioprosthetic valve close. **b** During right ventricular filling there is no movement of the right cranial valve leaflet (arrow), an impaired movement of the left cranial valve leaflet (dotted arrow) and a normal opening of the caudal leaflet. **c** Three-dimensional transesophageal echocardiography (right-atrial view) of the dysfunctional prosthesis with signs of leaflet degeneration (arrow) and the pre-existing pacemaker lead in place (dotted arrow)

Considering the history of three open-heart surgeries and the reduced general health condition, the patient was deemed at high surgical risk by the heart team (risk of in-hospital death according to EuroSCORE II 4.1%, risk of mortality according to STS Score for mitral valve replacement 3.5%). Consequently, a transcatheter valve-in-valve-in-valve implantation was scheduled. Based on pre-procedural CT analysis (area 544 mm^2^) we opted for another SAPIEN 3 29 mm prosthesis including pre-dilatation (Fig. 2).

**Fig. 2:**
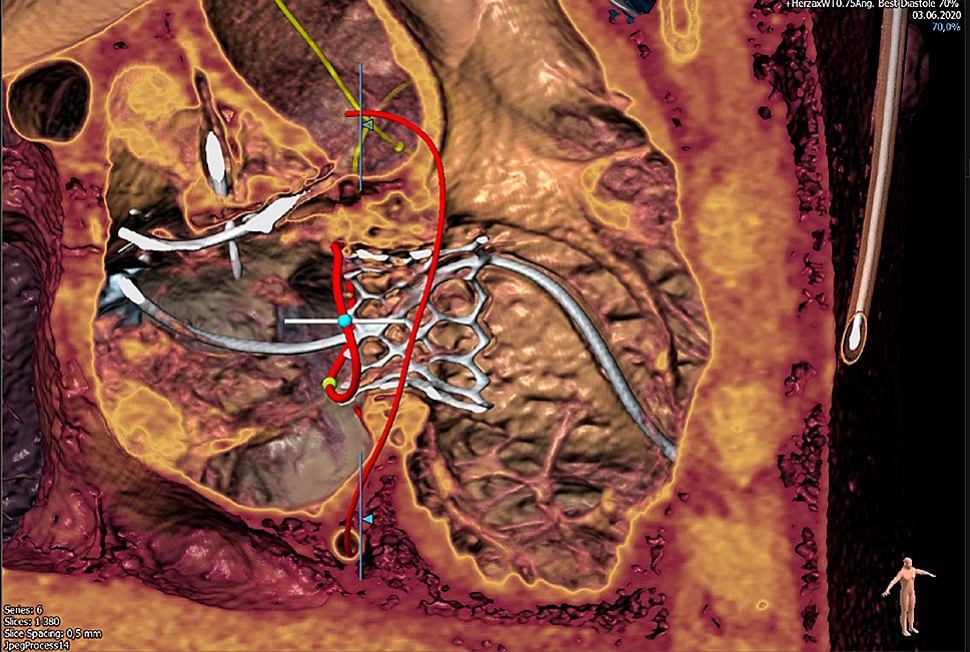
Pre-procedural CT analysis. The planning of the procedure and the decision regarding device sizing involved a pre-procedural 4D-CT which was analyzed in cooperation with AngioConsult GmbH. A perimeter derived diameter of 26.4 mm, an area derived diameter of 26.3 mm^2^ and an area of 544 mm^2^ led to the decision of using again an Edwards SAPIEN 3, 29 mm prosthesis

The procedure was performed under general anesthesia and mechanical ventilation. A J-wire was advanced into the right ventricle across the valve-in-valve tricuspid bioprosthesis (Fig. 3a) and exchanged for a Safari wire over an Amplatz Left catheter (upside down placing, Fig. 3b). Balloon predilatation was performed with a 23 mm Z-Med II balloon (NuMED Inc., New York, US) with rapid ventricular pacing. The SAPIEN 3 prosthesis was crimped inversely on the delivery catheter, then advanced over the femoral vein and retracted onto the balloon in the inferior vena cava. The prosthesis was advanced under fluoroscopic guidance using the flexion mechanism of the delivery catheter and positioned at the same height as the first implanted SAPIEN 3 prosthesis. Implantation was performed with rapid ventricular pacing (180 beats per minute) using the pre-existing trapped dual-chamber pacemaker (Fig. 3c, d). Intraprocedural transesophageal echocardiography confirmed good positioning of the valve without evidence of paravalvular regurgitation and a reduction of gradients (TV max PG 8.4 mmHg, TV MPG 4.0 mmHg). In the face of the accompanying optimal leaflet motion still elevated gradients are most likely due to a turbulent flow over the valve-in-valve-in-valve prosthesis. Postoperative pacemaker interrogation showed stable lead function.

**Fig. 3:**
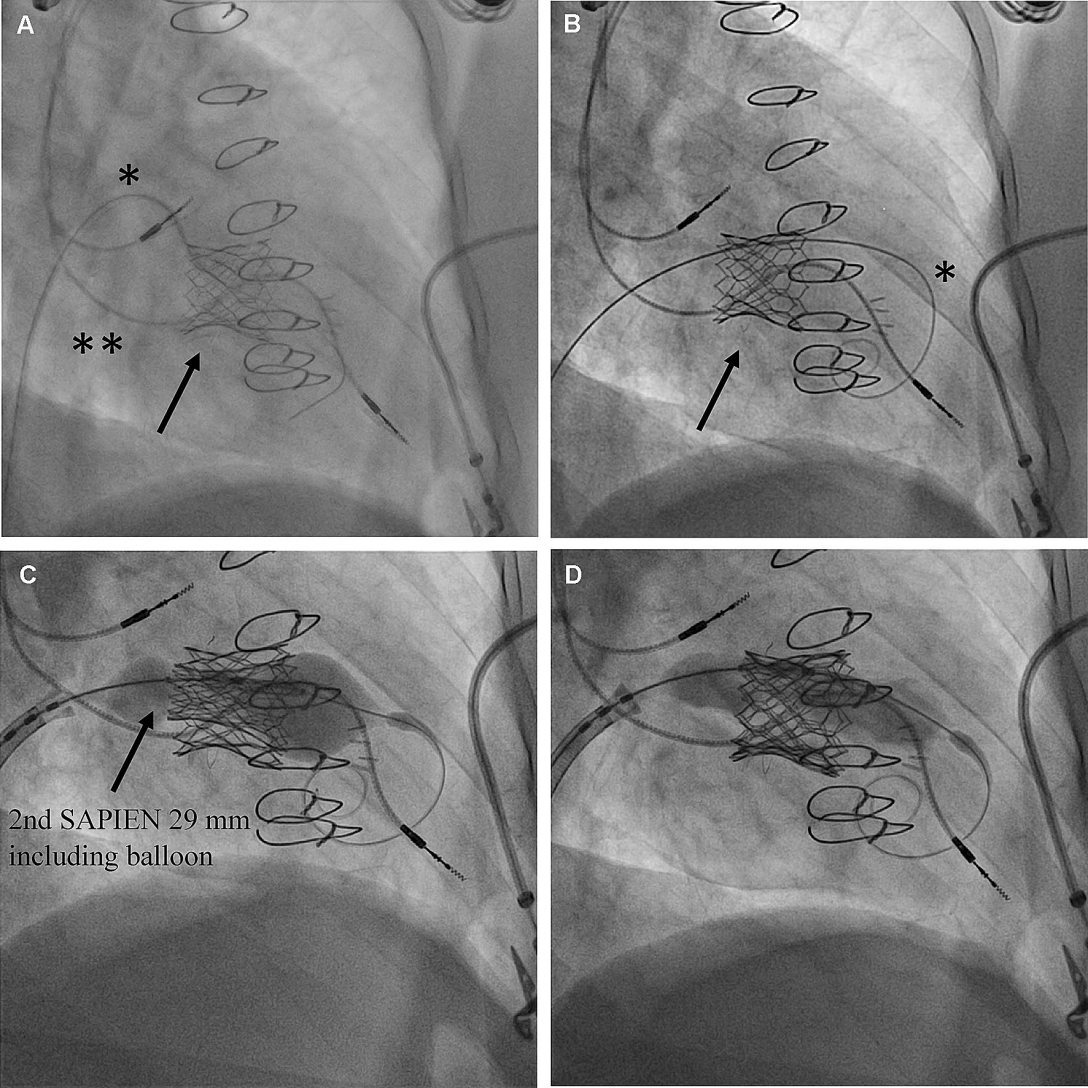
Transcatheter valve-in-valve-in-valve replacement in tricuspid position. **a** Coming from the left femoral vein an AL 1 catheter (*) crossed the initial SJM/SAPIEN prosthesis (arrow). The RV lead of the pre-existing dual-chamber pacemaker is already trapped between the surgical and TAVI prosthesis (**). **b** A safari wire (*) was used to establish a stable rail for antegrad valve delivery. **c** Balloon inflation during valve-in-valve-in-valve implantation of a second Edwards SAPIEN 3 29 mm during rapid ventricular pacing via dual-chamber pacemaker **d** Balloon deflation after implantation of a second Edwards SAPIEN 3 29 mm with optimal positioning

The postprocedural course was uneventful with a definite decline in symptoms and discharge in improved clinical condition 7 day post procedure. During the follow-up about 1 month after the valve-in-valve-in-valve replacement in tricuspid position the patient was in NYHA class II reporting that she was able to take 2–3 flight of stairs without recurrent syncopes.

Due to the complex anatomy of the tricuspid valve and concomitant right ventricular pathologies almost 30% of patients need surgical tricuspid valve replacement instead of repair [8, 9]. Both mechanical and biological prostheses are available for surgical tricuspid valve replacement without significant differences in terms of survival, reoperation, or prosthetic failure, but valve thrombosis seems to be more frequent after mechanical tricuspid valve replacement [13]. Hence, in current practice implantation of a bioprosthesis is preferred at many centers [2, 8].

For reasons still unclear, bioprostheses in tricuspid position are more prone to degeneration as bioprostheses implanted in aortic or mitral position. As a consequence, valve degeneration with the need for repeat intervention is a frequent finding in those patients [9]. Especially patients treated at young age with surgical tricuspid valve replacement (e.g., congenital heart defect [e.g., Ebstein anomaly], rheumatic valve disease, infective endocarditis) may require repeated valve replacements [14]. The risk of reoperation after implantation of a bioprosthesis increases at a steeper rate after 10 years with freedom from reoperation at 15 years ranging from 70 to 86% for mechanical valves and from 55 to 57% for bioprosthetic valves [11, 12]. Considering the mean age at tricuspid valve replacement ranging from 43.7 to 59.9 years [2, 8, 11, 12] and the high mortality rate of redo tricuspid valve replacement (about 26%) [1, 2], transcatheter tricuspid valve-in-valve replacement has become an important alternative to delay or avoid redo surgery.

Several studies in the recent past including an international, multicenter registry have shown that transcatheter tricuspid valve implantation after prior surgical repair or replacement is an effective and safe treatment option for high-risk/inoperable patients [3–7]. Here we describe the case of a highly symptomatic young woman who presented with dysfunction of the previously implanted transcatheter valve-in-valve bioprosthesis. Other causes for the severe dyspnea and recurrent syncope were previously ruled out. Although relatively young our patient already had undergone three open-heart surgeries which increased in fact the risk for redo surgery immensely. Compared to 2017 echocardiography at time of presentation to our hospital showed an increase in TV max PG (post-procedural 2017: 11.0 mmHg, pre-procedural 2020: 12.7 mmHg) and a nearly unchanged TV MPG (post-procedural 2017: 6.0 mmHg, pre-procedural 2020: 5.5 mmHg). Despite normal RV function reflected by RV FAC TAPSE was already reduced to 13 mm. In face of the accreting symptoms, the new evidence of a valve dysfunction, the already compromised TAPSE, and the young age of our patient we decided to perform valve replacement with the intention both to treat the patient due to the symptoms and to prevent any further decrement of RV function.

The intended procedure was, furthermore, complicated by a trapped pacemaker lead on which the patient was dependent due to complete AV block after the third open-heart surgery in 2011. In the largest series of transcatheter tricuspid valve replacements only 31 of 329 patients had a pre-existing transvenous pacemaker with a trans tricuspid valve lead. About 11% of the patients with intentional RV lead entrapment had lead complications [15]. In addition, there is only limited data showing that trapping of the ventricular lead between the surgically implanted valve and the transcatheter bioprosthesis may be safe [16, 17]. Still, the probability of acute damage and subsequent dysfunction of the ventricular lead during balloon valvuloplasty and valve implantation had to be considered. Therefore, the function of the dual chamber pacemaker was monitored continuously during implantation. In case of acute pacing lead dysfunction, both external pacing and coronary sinus pacing catheters were prepared and ready. Discussed bail out strategies for a dysfunctional permanent RV pacing lead were (1) the implantation of a His bundle pacer, (2) the implantation of a leadless pacer (Micra), or (3) surgical implantation of an epicardial RV pacing lead. However, postoperative interrogation of the device showed a stable lead function which is in line with the previous reports of unaffected lead function after valve-in-valve implantation.

Summarized, we here report for the first time that valve-in-valve-in-valve replacement in tricuspid valve position is a feasible and safe alternative to delay or avoid redo surgery in patients with failing transcatheter implanted valve-in-valve prostheses. In addition, a pre-existing RV pacemaker lead does not seem to be compromised by an additional TAVI prosthesis; however, bail out strategies for a dysfunctional ventricular lead should be prepared and applicable during the procedure.
